# Medical News and Miscellany

**Published:** 1891-10

**Authors:** 


### Medical News and Miscellany.

---

**Dr. J. W. Matthews**, of Austin, is at the New York Polyclinic.

---

**Dr. E. G. Adams**, late of Caledonia, has removed to Garrison, and entered into a copartnership with Dr. Quillain of that city.

---

**Dr. G. D. Lane**, of Camp Colorado, is taking a special course at the Medical Department, University of Louisville, and requests the JOURNAL sent him there during the session.

---

**Dr. J. H. Reuss**, of Cuero, the handsome young surgeon of the 3rd Texas Regiment, T. V. G., has gone to New York, to take a post graduate course at one of the many colleges there.

---

**Dr. C. M. Scogin**, who removed some time ago from Throckmorton to Albany, has returned to his old home, and, if reports be true to his "first love." We expect to be favored with "cards."

---

**Our notorious Dallas contemporary** advises all of his

"friends who have heavy legal business on hand," to see the Honorable Wudley G. Dooten—his own attorney—"before employing '*council*'."

---

ERRATUM.—Omit *Morgagni* at beginning of Beall's paper. Name belonged to a Latin quotation in original, stricken out in copy sent us. This quotation is from a letter from Wilson, of Philadelphia.—ED.

---

**A New Firm.**—The JOURNAL learns that Dr. F. E. Young, of Brownwood, and Dr. R. E. Moss, of Mexia, have formed a copartnership, and removing to San Antonio, will open a sanitorium at that place. Success to them.

---

**The Chair of Surgery** in the Texas Medical college will be filled by Prof. J. E. Thompson, of Manchester, a most brilliant and accomplished surgeon, who comes endorsed by the highest authority, medical and surgical, in England, Germany, France and Scotland.

---

**Dr. D. B. McGee**, of Brownwood, has been appointed County Physician, and Surgeon to the Ft. Worth and Rio Grande railroad. Read report of his case of "circumscribed dropsy"—and write to him about it, if you ever heard of anything like it; it is a remarkable case.

---

**Dr. J. M. T. Finney**, of Baltimore, the young man whom the Board of Regents, Texas University, honored by offering him the appointment to the chair of Surgery in the Texas Medical College, has declined the offer, preferring to remain at the Johns Hopkins in the character of an assistant. *Degustibus non*, etc.

---

**Faculty Organized.**—At a meeting of the faculty of the Texas Medical College (Medical Department, University of Texas) held in Galveston, October 1 inst., Dr. J. F. Y. Paine, Professor of Obstetrics and Gynecology, was elected Dean, and Dr. Seth M. Morris, Professor of Chemistry, Secretary. The preliminary course begun on the 5th, regular course on the 12th inst.

---

**Struck by Lightning.**—During one of the afternoon thunderstorms that visited Austin during September, Dr. H. B. Hill's residence was struck by lightning, and a chimney was demolished. The Doctor's baby, a two-year-old daughter, was standing near the grate, and was thrown half across the room without sustaining any injury beyond a fright; but the parents were worse frightened.

**Death of an Old Texas Physician: Dr. G. F. Weisselburg**—One of the oldest practitioners of Austin, died at his home in Austin, Saturday, September 19th. He was 67 years of age: was a member of the Knights of Honor, and of the Legion of Honor, and was buried by those orders in the city cemetery, in Austin, on Sunday, the 20th ult. the Episcopal service being read by Rev. T. B. Lee, of St. David's church, and that of the Lutheran church by the minister of that church. His youngest daughter is the wife of our late townsman, Prof. B. E. Hadra, M. D.

**Death of Dr. T. S. Burke.**—Dr. Thomas Somervel Burke, of Corpus Christi, died at his home September 21, ult., after a long illness. He held the position of quarantine physician at Aransas Pass many years, and only retired upon the change of administration. Since which time he had been confined to his bed. Dr. Burke was a man of fine qualtities, a physician of more than ordinary skill and ability, and was held in high eeteem by all who knew him. His son, H. S. Burke, has begun the study of medicine; may he make as good a physician as his lamented father was. The family have the sympathy of the profession and the sincere condolence of the JOURNAL

**A Good Location** for a physician who is willing to pay $200 for drugs and good will of the only M. D. in the place.

Moore is a station on the Santa Fe railroad, nine miles south of Oklahoma City, and nine miles north of Norman, the county seat of Cleveland county. It is surrounded by a class of good farmers, who are mostly from Texas, Arkansas and other Southern States.

The practice from here can be made to pay $2000 a year, or more, by putting in a small stock of drugs. Office rent, $4 per month. None but a graduate need apply.

Address: Dr. W. J. FARIS,
Moore, Cleveland Co., O. T.

**Dr. Bransford Lewis'** new journal, to be published at St. Louis, (the initial number Jan. 1, '92), is to be called the "Fortnightly M. D.'' The number of physicians who responded to his unique advertisement, which appeared in this, and other journals, was 39,001. Those who guessed correctly the name of the journal as signified by the peculiar design of the advertisement—numbered 6,422. We gave it up at once. The crescent, the Doctor explains, ''stood for fortnightly,'' and the skeleton fingers shaped into F. M. D. "in deaf and dumb language,'' indicated the name. But we venture to "guess'' that the Doctor will get out a good journal, whether fortnightly or not. But *un-fortnately* he will have a goodmany dead head subscribers.

---

Died—In Austin, Texas, August 31, 1891, Mrs. ELLA DENTON GIVENS, daughter of Dr. A. N. Denton, of Austin — late Superintendent State Lunatic asylum.

Mrs. Given was a most estimable lady, one of rare cultivation and refinement, and her early death is mourned by an extensive circle of admiring and attached friends. By her gentle demeanor and rare Christian virtues, she won the hearts of all, especially the poor, to whom she was a ministering angel. She was a very accomplished musician, and her presence in social gatherings lent a charm that will now be sadly missed.

She was born April 18, 1864, and on the 13th of February, 1885, she was married to Dr. Jas. G. Given, a talented young physician, who died, leaving her one child, now a bright boy. She had been for some time a great sufferer from renal calculi, but bore her affliction with fortitude, till finally a desperate operation was necessary—the one chance to save her life and relieve the dreadful suffering. To this she submitted with Christian resignation, but sank under the shock and hemorrhage, and passed away. Dr. Denton has the sympathy of the entire community in his affliction.

---

The Medical Department University of Texas.—The first session of the new Texas Medical College was formally opened on the 12th inst., at 12 m., by Prof. J. F. Y. Paine, M. D., Dean of the Faculty, in an able and interesting address. Lectures began on the morning of the 13th to a class of twenty sudents.

The building is not yet quite finished—in certain details, but will be shortly. Dr. Seth M. Morris, the Professor of Chemistry, was elected Secretary of the Faculty.

The Professor of Surgery, Dr. J. E. Thompson, of Manchester, England, had not arrived up to the opening of the session, but is daily expected. Dr. Wm. Keiller, of Edinburgh, the Professor of Anatomy, whose election to that chair was announced in the JOURNAL last month, is present. He is a man of very small stature, but is said to be a master of his department of science. Dr. Smith, the Pathologist, selected from Philadelphia, is at his post also, and everything is moving along smoothly. Dr. Clopton, the Physiologist, late of Jefferson, Texas, was in Austin on the 10th, in company with Dr. Thompson, of the Board of Regents, and favored the JOURNAL office with a call. They came up on business for the Faculty, to confer with President Wooten.

---

"**When Lovely Woman Stoops to Folly.**"—The doctor sees many phases of human life not visible to anyone else, and alas he sees less of the bright side than of the dark. He sees the sorrow, and often depravity that constitute the skeleton in many a closet; he is the custodian of numerous secrets, and the forlorn hope of *les miserables.*

One of our most esteemed and honorable practitioners, —a man of large sympathy and strictest integrity—received the following note from an erring daughter of Eve, who, having "learned too late that men betray," vainly hoped that there was a "power" that can "sooth her melancholy," or that the "art" of medicine "can wash her guilt away." Judging from the literary aspect of the epistle the writer would not properly come under the head of "lovely woman;" that is, if her person be not of a higher order of excellence than her education;—but —who can tell? Not every fair face is the reflex of scholastic finish, nor every graceful form the result of school-house discipline. Cleopatra, it is to be presumed, would have capitalized the wrong words, had she been inditing a *billet doux* to Mr. Anthony, and yet, what a fuss was created by her beauty, and what a fight for her favors !

And, by the bye,—"unmarriage;"—here is a suggestion: why not "unmarriage" to express the idea that the condition of this poor creature was not the result of the "holy state of matrimony?" It is brief, expressive, and to the point. That very word "marry" is arbitrary and unsatifactory. A man marries the object of his devotion, a girl marries her lover; and the preacher "marries" them both, eh? Not so. The late good old Bishop Green, of Mississippi, coined, and always used the word "marrify" or "marrified" to express the part he performed in uniting the customary "two hearts." He repudiated indignantly the assertion that he had "married a couple," or had "married two people;''—he said he couldn't do it, but he could perform that rite of the church which made the parties man and wife; he made the married,—he therefore *marri-fied* them.

But, to the letter; it ran thus, *verbatim et literatim:*

for unmarriage

Mr Dr ——, Please send me Medicine for Woman Disstroy When in Family Ways for a few days sence.

Also to Prevent of geting in any such as Family Ways.

Please give stron with full Directions as to be True in its Disstroy and ferther appearence

I sent the Money With letter to Pay for the same Please give me few instructions if think necssary.

